# DICE: A Drug Indication Classification and Encyclopedia for AI-Based Indication Extraction

**DOI:** 10.3389/frai.2021.711467

**Published:** 2021-08-02

**Authors:** Arjun Bhatt, Ruth Roberts, Xi Chen, Ting Li, Skylar Connor, Qais Hatim, Mike Mikailov, Weida Tong, Zhichao Liu

**Affiliations:** ^1^ Division of Bioinformatics & Biostatistics, National Center for Toxicological Research, Food and Drug Administration, Jefferson, AR, United States; ^2^ Dartmouth College, Hanover, NH, United States; ^3^ Brody School of Medicine, East Carolina University School of Medicine, Greenville, NC, United States; ^4^ ApconiX Ltd, Alderley Edge, United Kingdom; ^5^ Department of Biosciences, University of Birmingham, Birmingham, United Kingdom; ^6^ Office of Translational Sciences, Center for Drug Evaluation and Research, US FDA, Silver Spring, MD, United States; ^7^ Office of Science and Engineering Labs, Center for Devices and Radiological Health, U.S. Food and Drug Administration, Silver Spring, MD, United States

**Keywords:** natural language processing, deep learning, artificial intelligence, transformers, drug indication

## Abstract

Drug labeling contains an ‘INDICATIONS AND USAGE’ that provides vital information to support clinical decision making and regulatory management. Effective extraction of drug indication information from free-text based resources could facilitate drug repositioning projects and help collect real-world evidence in support of secondary use of approved medicines. To enable AI-powered language models for the extraction of drug indication information, we used manual reading and curation to develop a **D**rug **I**ndication **C**lassification and **E**ncyclopedia (DICE) based on FDA approved human prescription drug labeling. A DICE scheme with 7,231 sentences categorized into five classes (indications, contradictions, side effects, usage instructions, and clinical observations) was developed. To further elucidate the utility of the DICE, we developed nine different AI-based classifiers for the prediction of indications based on the developed DICE to comprehensively assess their performance. We found that the transformer-based language models yielded an average MCC of 0.887, outperforming the word embedding-based Bidirectional long short-term memory (BiLSTM) models (0.862) with a 2.82% improvement on the test set. The best classifiers were also used to extract drug indication information in DrugBank and achieved a high enrichment rate (>0.930) for this task. We found that domain-specific training could provide more explainable models without performance sacrifices and better generalization for external validation datasets. Altogether, the proposed DICE could be a standard resource for the development and evaluation of task-specific AI-powered, natural language processing (NLP) models.

## Introduction

Drug labeling contains an ‘INDICATIONS AND USAGE’ section that provides vital information to support clinical decision making and regulatory management. The primary role of drug indications is to enable health care practitioners to readily identify appropriate therapies for patients and support clinical decision making (Sohn and Liu, 2014). The information on drug indication is part of the required information in FDA approved drug labeling and guides the content and format of labeling of human prescription drugs and biological products [21 CFR 201.57(c) (2)]. Drug indications also provide guidance for facilitating clinical knowledge management and play an essential role in enabling the secondary use of electronic medical records (EMRs) for clinical-based translational research. Besides the primary drug indication approved for the drug, information on off-label uses and repurposing opportunities, or alternative uses of drugs, are common within biomedical-related data resources such as scientific literature, patents, public health forums, and pharmacological, biomedical, or drug labeling databases (Salmasian et al., 2015; Delavan et al., 2018). Furthermore, indication information extraction is also a regulatory requirement for creating the highlights section of the Physician Labeling Rule (PLR) labeling, which provides concise information for public health practitioners, patients and drug reviewers (https://www.fda.gov/drugs/laws-acts-and-rules/prescription-drug-labeling-resources). Thus, developing an effective approach to facilitate the mining of drug indication information from free text-based resources is an important task for biomedical natural language processing (NLP).

Some attempts to extract drug indications from free text-based documents have been undertaken, mainly based on the combination of named entity recognition (NER) approaches with conventional machine learning algorithms (Fung et al., 2013; Khare et al., 2014; Khare et al., 2015). One example is a two-step strategy for drug indication extraction proposed by Khare et al. 2014. Here, disease terminology is extracted from over 500 drug labels using a MetaMap tool with the Unified Medical Language System (UMLS)-based disease lexicon as the control vocabulary (Aronson, 2001). Then, a binary support vector machine (SVM) classifier is implemented to distinguish drug indication from other information such as adverse drug reactions, yielding an 86.3% F1 measure (the measure of a model’s accuracy) for the indication extraction task, representing a 17% improvement over baseline approaches. With advances in AI-powered NLP, new approaches have been developed, which may provide additional performance improvements in the task of drug indication extraction. Artificial intelligence (AI)-powered language models such as transformers have achieved greater improvement compared to other approaches in various NLP tasks (Vaswani et al., 2017a; Devlin et al., 2018). Several biomedical-based BERT models (i.e., BioBERT, SciBERT, and clinicalBERT) have been developed for domain-specific tasks such as biomedical named entity recognition (NER) (Beltagy et al., 2019; Huang et al., 2019; Lee et al., 2020). Disease entity recognition corpora, such as the NCBI disease corpus, have become widely established sources for developing AI-based NER approaches (Doğan et al., 2014). However, the lack of large corpora for disease information classification hampers AI-based NLP development, and efforts to address this gap are urgently needed (Khare et al., 2015).

Based on guidance for industry on the ‘INDICATIONS AND USAGE’ section of Labeling for Human Prescription Drug and Biological Products, content should be concise but unambiguous. The information in the ‘INDICATIONS AND USAGE’ section should readily allow the identification of approved indication(s) and reflect current scientific evidence. Furthermore, indication terminology should be standardized, clinically relevant, scientifically valid, and easily understandable. Also, this information should be consistent within/across drug and therapeutic classes to aid the indexing of indications in electronic drug databases and medical information systems. Drug indication information often comprises mixed information such as age group, subpopulations, classifications such as adjunctive or concomitant therapy, specific tests/diagnoses, and other disease conditions or clinical manifestations. Thus, drug labeling is a great resource for drug indication classification, facilitating the development of AI-based NLP models, and further improving drug indication information extraction.

We developed a five-category **D**rug **I**ndication **C**lassification and **E**ncyclopedia (DICE) based on FDA approved human prescription drug labeling to facilitate the development of AI-based NLP approaches for enhanced drug indication extraction from free text-based document resources. The DICE scheme categorizes the >7,000 sentences in the ‘INDICATIONS AND USAGE’ section into five classes, including indication, contraindication, side effect, usage instruction, and clinical observations. To verify the utility of DICE, we developed nine different AI-based classifiers, including 4-word embeddings-based Bidirectional long short-term memory (BiLSTM) models and five transformer-based language models. The model performances were comprehensively assessed based on a test set and an independent validation set. Some critical questions such as the benefit of domain-specific training for AI-based NLP were also investigated. Furthermore, the model explainability was discussed for real-world applications.

## Materials and Methods

**FIGURE 1:**
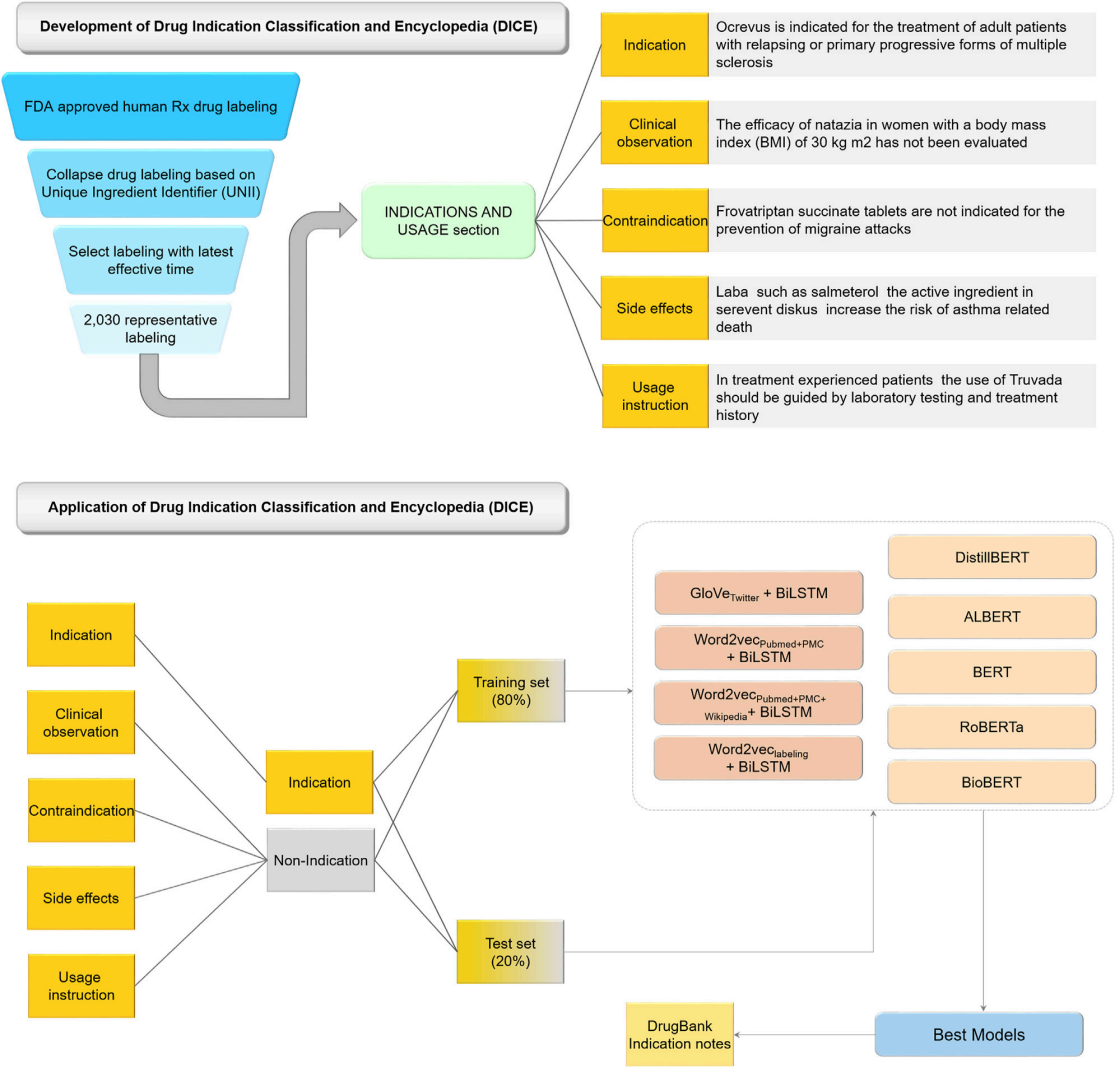
Workflow of the study.

Figure 1 illustrates the workflow of the study. The study consisted of two components: DICE development, and AI-powered indication classification model development based on DICE.

### Drug Indication Classification and Encyclopedia Development

To curate an indication classification corpus, we employed US Food and Drug Administration (FDA)-approved drug labeling. Drug labeling, also known as the package insert or prescribing information, accompanies every FDA approved medicine as required under the US Code of Federal Regulations (21 CFR 201.56). Drug labeling is submitted by the manufacturer and approved by FDA and includes a rich source of information on safe and effective drug usages. There are more than 80 sections embedded in a drug labeling document (Fang et al., 2020). Among the labeling sections, the INDICATIONS AND USAGE section aims to enable health care practitioners to readily identify appropriate therapies for patients by clearly communicating the drug’s approved indication(s) (https://www.fda.gov/media/114443/download). The ‘INDICATIONS AND USAGE’ section mainly contains information such as “1) The disease, condition, or manifestation of the disease or condition (e.g., symptoms) being treated, prevented, mitigated, cured, or diagnosed;” and “2) When applicable, other information necessary to describe the approved indication (e.g., descriptors of the population to be treated, adjunctive or concomitant therapy, or specific tests needed for patient selection).” Since the ‘INDICATIONS AND USAGE’ section contains such a variety of information, it is imperative to develop an indication classification corpus for automatic indication extraction.

Specifically, we extracted a list of FDA approved drug labels by using a search query “human Rx” under labeling type in the FDALabel databases (version 2.5, https://nctr-crs.fda.gov/fdalabel/ui/search) (Mehta et al., 2020). Consequently, we obtained queried results with summary information of human prescription (Rx) drug labeling. To obtain ‘INDICATIONS AND USAGE’ sections for a unique list of human prescription drug labels, we implemented the following strategy: 1) collapse the labeling with the same Unique Ingredient Identifier (UNII); 2) select the labeling with latest effective time as the representative one (i.e., XML file) for each collapsed labeling; 3) extract ‘INDICATIONS AND USAGE’ section from XML file based on Logical Observation Identifiers Names and Codes (LOINC) for Human Prescription Drug and Biological Product Labeling (https://www.fda.gov/industry/structured-product-labeling-resources/section-headings-loinc). The LOINC code for INDICATIONS AND USAGE section is “34067-9” (Figure 1).

To manually annotate the information in the ‘INDICATIONS AND USAGE’ sections into different categories, we developed a five-class indication classification scheme (Figure 1). We split the extracted ‘INDICATIONS AND USAGE’ sections into sentences. Each of the 7,231 sentences were placed into one of five categories, namely indication, contraindication, side effect, usage instruction, and clinical observations. Assignments were based on predefined keywords and *a priori* knowledge. Three experienced, expert pharmacologists carried out the manual annotations independently and a consensus assignment was selected for indication information classification.

### AI-Powered Indication Extraction Model Development

For the purposes of indication extraction, the extracted 7,231 sentences assigned with the category ‘indication’ were considered as positives, and sentences assigned to any of the other four categories were considered negative. The 7,231 curated sentences were divided into the training and test sets with an approximate ratio of 80:20. Consequently, we obtained 5,785 sentences and 1,446 sentences for the training and test sets, respectively. Two types of deep learning models were developed, including word embedding-based BiLSTM models, and transformer-based language models.

#### Preprocessing

We implemented the following procedure to preprocess the sentences: 1) the sentences were tokenized; with stripping of punctuation, digits, and words with less than two characters; 2) stop word removal; and 3) lemmatization.

##### Word Embeddings

Word embedding is a set of language modeling and learning techniques in NLP to map words or phrases from a vocabulary to a numeric vector representation. In this study, we used two types of word embeddings including Word2vec (Mikolov et al., 2013a; Mikolov et al., 2013b) and Glove (Pennington et al., 2014).

Word2vec is a shallow neural network framework (i.e., continuous bag-of-words (CBOW) and continuous skip-gram) used to estimate continuous vector representations of words from large text corpora (Mikolov et al., 2013a; Mikolov et al., 2013b). The generated word embeddings position words with common contexts close to one another. Word2vec has been used widely in NLP tasks such as semantic relationship extraction (Chen et al., 2018), text classification (Jang et al., 2019), and sentiment analysis (Rezaeinia et al., 2019). In this study, we use three pretrained domain-specific word2vec models, including Word2vec with PubMed and PMC (i.e., Word2vecPubmed + PMC), word2vec with PubMed, PMC, and Wikipedia (i.e., Word2vecPubmed + PMC + Wikipedia), and word2vec with FDA approved human prescription labeling (Word2veclabeling). The Word2vecPubmed + PMC and Word2vecPubmed + PMC + Wikipedia (200-dimension vector models) were downloaded from https://bio.nlplab.org/(Moen and Ananiadou, 2013). We developed the pre-trained Word2veclabeling by using the human labeling documents described above. The in-house implementation of word2vec was consistent with the PubMed corpus; briefly, the implementation used the skip-gram model with a window size of 5, hierarchical SoftMax training, and a word subsampling threshold of 0.001 to create 200-dimensional vectors. The training was conducted using the Python Gensim package (version 0.6.0).

We also employed another well-known word embedding technique (i.e., GloVe 200-dimension vectors), which, when applied to aggregated global word-word co-occurrence statistics from a corpus, generate word vector representation (Pennington et al., 2014). Specifically, the pretrained GloVe model with 2 billion Twitter corpus was employed as the general domain specific word embedding (i.e., GloVeTwitter); this corpus can be downloaded from https://nlp.stanford.edu/projects/glove/.

##### Bidirectional Long Short-Term Memory

To better understand the framework and theory behind the BiLSTM, we provide a simple introduction on the Recurrent Neural Network (RNN) and Long Short-Term Memory (LSTM). An RNN is a set of artificial neural networks for sequential and time-series data. Unlike conventional neural networks, RNNs adopt recurrent hidden states to store previous inputs and leverage sequential information of the previous inputs to estimate the next element in the sequence. In theory, RNNs are able to leverage previous sequential information for arbitrarily long sequences. In practice, however, due to RNNs memory limitations called “vanishing gradients”, the length of the sequential information is limited to only a few steps back (Hochreiter et al., 2001).

Hochereiter and Schimdhuber (Hochreiter and Schmidhuber, 1997) proposed the LSTM model, which is a gated RNN intended to solve the “vanishing gradients” problem and greatly expand RNNs applications for long sequence data (Gers et al., 1999). The LSTM cell consists of four components (i.e., input gate, memory cell, forget gate, and output gate) to remember information over a longer period of time and thus enable reading, writing, and deleting information from the cell’s memory. The forget gate makes the decision of preserving/removing the existing information, the input gate specifies the extent to which the new information will be added into the memory, and the output gate controls whether the existing value in the cell contributes to the output (Siami-Namini et al., 2019). The deep-BiLSTMs are an extension of the described LSTM model above, in which two LSTMs are applied to the input sequence (i.e., forward layer) and reverse of the input sequence (i.e., backward layer) (Schuster and Paliwal, 1997). Applying the LSTM twice leads to the enhanced learning of long-term dependencies and thus improves the accuracy of the model.

Supplementary Figure S1 illustrates the proposed BiLSTM model infrastructure for indication classification. The processed sentences were vectorized by the different word embedding techniques described above; the now vectorized sentences were then fed into bidirectional LSTM layers and a dense layer, followed by a flattened layer and a dense layer. The output layer is a probabilistic value of sentences belonging to the indication information category. Specifically, we used a learning rate of 0.001, Rectified Linear Units (ReLU) activation, and an Adagrad Optimizer. The optimizer was chosen due to its suitability for training on sparse data and its ability to perform more informed gradient-based learning.

#### Transformer-Based Language Models

To further investigate the performance of advance AI-powered NLP approaches on indication classification, we employed the Bidirectional Encoder Representations from Transformers (BERT) (Vaswani et al., 2017a; Devlin et al., 2018) and its derivatives including a distilled version of BERT (DistilBERT) (Sanh et al., 2019), A Lite BERT (ALBERT) (Lan et al., 2019), a Robustly Optimized BERT Pretraining Approach (RoBERTa) (Liu et al., 2019), and a pre-trained biomedical BERT (BioBERT) (Lee et al., 2020).

BERT is a transformer that learns contextual bidirectional representations from an unlabeled, large corpus of documents by using two training strategies: Masked Language Model (MLM) and Next Sentence Prediction (NSP) (Vaswani et al., 2017b; Devlin et al., 2018). In the MLM, a randomly selected 15% of words in a sequence are replaced with a [MASK] token, and the model aims to estimate masked words, based on the context provided by unmasked words. In the NSP, the model aims to utilize the pairs of sentences as inputs and predict the sequence order in the original documents. The BERT model has achieved state-of-the-art performance on diverse sets of NLP tasks (e.g., text classification, named entity recognition) while requiring only minimal task-specific architectural modification (i.e., fine-tuned layers).

Two condensed BERT models, DistillBERT and ALBERT, were proposed to overcome the obstacle of long training times. DistilBERT uses a technique called distillation, which approximates the BERT from the large neural network to a smaller one. By learning from the distilled version of BERT, DistilBERT retained about 97% performance while using only half as many parameters as the original BERT (Sanh et al., 2019). One of the key optimization functions used for posterior approximation in DistilBERT is Kulback Leiber (K-L) divergence to condense the network size while maintaining performance. ALBERT is a light version of BERT, which employs two techniques to reduce the parameters, including Factorized Embedding Parametrization and Cross-layer Parameter Sharing (Lan et al., 2019). Additionally, a self-supervised objective is proposed for sentence order prediction to further improve performance, addressing the suboptimal performance of the NSP task from BERT.

RoBERTa is an updated version of BERT that improves the pretrained optimization process (Liu et al., 2019). First of all, RoBERTa uses a much larger set of training data (161 GB) for pretraining to increase the model’s generalization ability. Secondly, instead of the static masking pattern used in the MLM model, RoBERTa introduced a dynamic masking pattern to avoid same training mask for each training instance. Also, the RoBERTa model developed training objectives to enhance NSP model performance. Moreover, RoBERTa trained on longer sequences than BERT to further improve performance.

It was observed that generic pretrained transformer models may not work very well in conjunction with specific domain data. To fill this gap, BioBERT, a domain-specific BERT model, was proposed by training the BERTbase model on large biomedical corpus including PubMed abstracts and PMC full text (Lee et al., 2020). The BioBERT model outperformed BERTbase on some domain-specific tasks such as biomedical named entity recognition (NER), and bio-Questions and answering with a 0.51–9.61% absolute improvement.

BERT-like models are designed as pre-trained deep bidirectional representations from unlabeled text by jointly conditioning on both left and right context in all layers. They are then fine-tuned with an additional output layer to create models for a wide range of tasks, such as question answering and language inference, without substantial task-specific architecture modifications. The fine-tuned base models of transformers were used in this study for the binary classification task for indication recognition. An important difference is that these models used their native tokenizers, which utilized sub-word tokenization (e.g., WordPiece) where larger words may be broken down to map to token(s), compared to the cruder tokenization implemented with the simpler model.

### Model Performance Evaluation

To train the model and measure model performance, we employed area under the receiver operating characteristic (ROC) curve analysis, which demonstrates the performance of the classification model by plotting the true predictive rate (TPR) against the false positive rate (FDR). We calculated the area under the ROC curve (AUC) for each model described above. We also used seven other performance metrics including Matthews correlation coefficient (MCC), accuracy, sensitivity, specificity, precision, negative predictive value (NPV), and F1-score for further evaluation of model performance by using the following confusion matrix and formulas

$$MCC=\frac{TP*TN-FP*FN}{\sqrt{\left(TP+FP\right)*\left(TP+FN\right)*\left(TN+FP\right)*\left(TN+FN\right)}}$$ (1)

$$accuracy=\frac{TP+TN}{TP+TN+FN+FP}$$ (2)

$$sensitivity=\frac{TP}{TP+FN}$$ (3)

$$specificity=\frac{TN}{TN+FP}$$ (4)

$$PPV=\frac{TP}{TP+FP}$$ (5)

$$NPV=\frac{TN}{TN+FN}$$ (6)

$$F1=\frac{2TP}{2TP+FP+FN}$$ (7)

**Table:** 

		Actual class	
		Indication (positive)	Non-indication (negative)
Predicted class	Indication (positive)	True positive (TP)	False positive (FP)
	Non-indication (negative)	False negative (FN)	True negative (TN)

### External Validation

To further investigate real-world applications of the developed indication classification model, we applied the best-performing models to indication descriptions in the DrugBank database. The indication information in DrugBank is a relatively concise description of the indication and usage of approved or investigational drugs (Wishart et al., 2018). Specifically, the DrugBank (version 5.1, downloaded on April 02, 2021) XML file was downloaded via https://go.drugbank.com/releases/latest. We developed an in-house script to extract the drug indication information from DrugBank XML file. Consequently, a list of 3,976 indication descriptions in DrugBank were extracted to further verify our developed model.

### Visualization

To investigate the discrimination powers of different word embeddings or sentence embeddings yielded from the transformers models used in this study, we employed t-distributed stochastic neighbor embedding (t-SNE) (Hinton and Roweis, 2002). t-SNE is a non-linear dimension reduction method. With t-SNE, the algorithm calculates the similarity in both high dimensional space and low dimensional space. Next, the similarity difference in both spaces is minimized using an optimization method such as gradient descend.

### Data and Code Availability

We developed a GitHub webpage (https://github.com/arjun-bhatt/TransformersIndicationExtraction) to share the source code and curated drug indication corpus. Specifically, all the code script is developed under Python 3.6. The BiLSTM model is based on tensorflow version 1.12.3. The transformers models were based on Huggingface package version 3.02 and its backend is tensorflow version 2.3.0 and PyTorch version 1.5.1. t-SNE was implemented by using Python Scikit-learn package version 0.23.2.

## Results

### Drug Indication Classification and Encyclopedia

**FIGURE 2:**
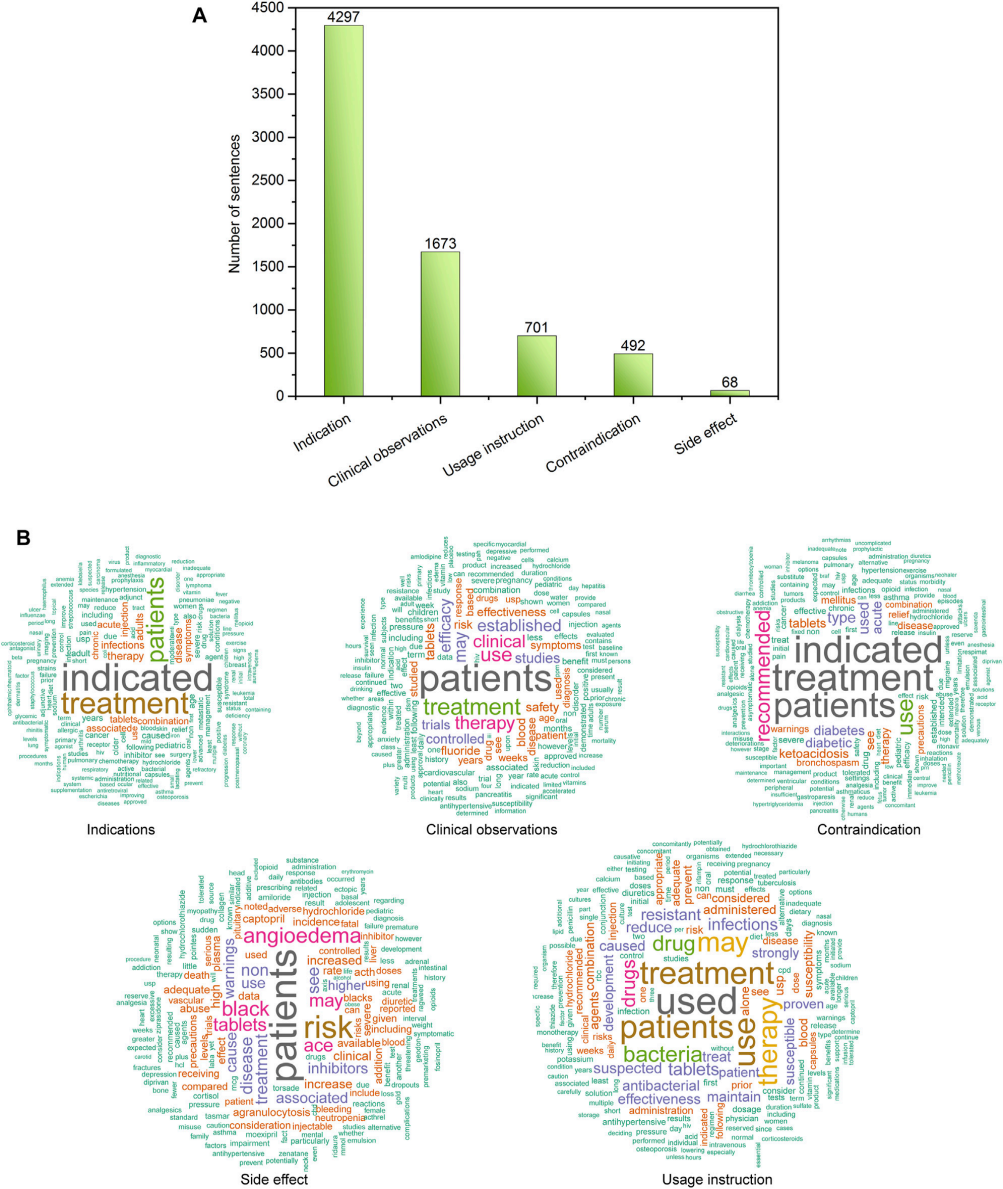
**(A)** Distribution of sentences in the proposed DICE scheme; **(B)** word cloud of the sentences in each defined DICE category.

Figure 2A illustrates the distribution of 7,321 sentences in the proposed drug indication classification scheme. To curate a high-quality drug indication classification, three pharmacologists manually read the sentences and assigned them into five predefined categories including indication, non-indication miscellaneous, contraindication, side effect, and usage instruction. Based on consensus manual annotation results, the 7,231 sentences were categorized into 4,297 indication, 1,673 clinical observations, 701 contraindication, 492 usage instructions, and 68 side effects (supplementary Table S1). Figure 2B depicts the most frequent words in each indication classification category using word clouds. For example, the top five key words in the Indication category were “indicated”, “treatment”, “patients”, “therapy”, and “disease”, respectively (supplementary Table S2). To develop an indication recognition classifier, we used the 4,297 indication as positives, and 2,934 combined sentences from the other categories as negatives, yielding a ratio between positives and negatives of 1.46. Then, we randomly split the 7,231 into a training set (80%) and a test set (20%). Accordingly, the training set (i.e., 5,785 sentences) consisted of 3,452 positives and 2,333 negatives (P/N ratio = 0.596), and the test set (i.e., 1,446 sentences) consisted of 845 positives and 601 negatives (P/N ratio = 0.584).

### Word Embedding-Based Bidirectional Long Short-Term Memory Models

**FIGURE 3:**
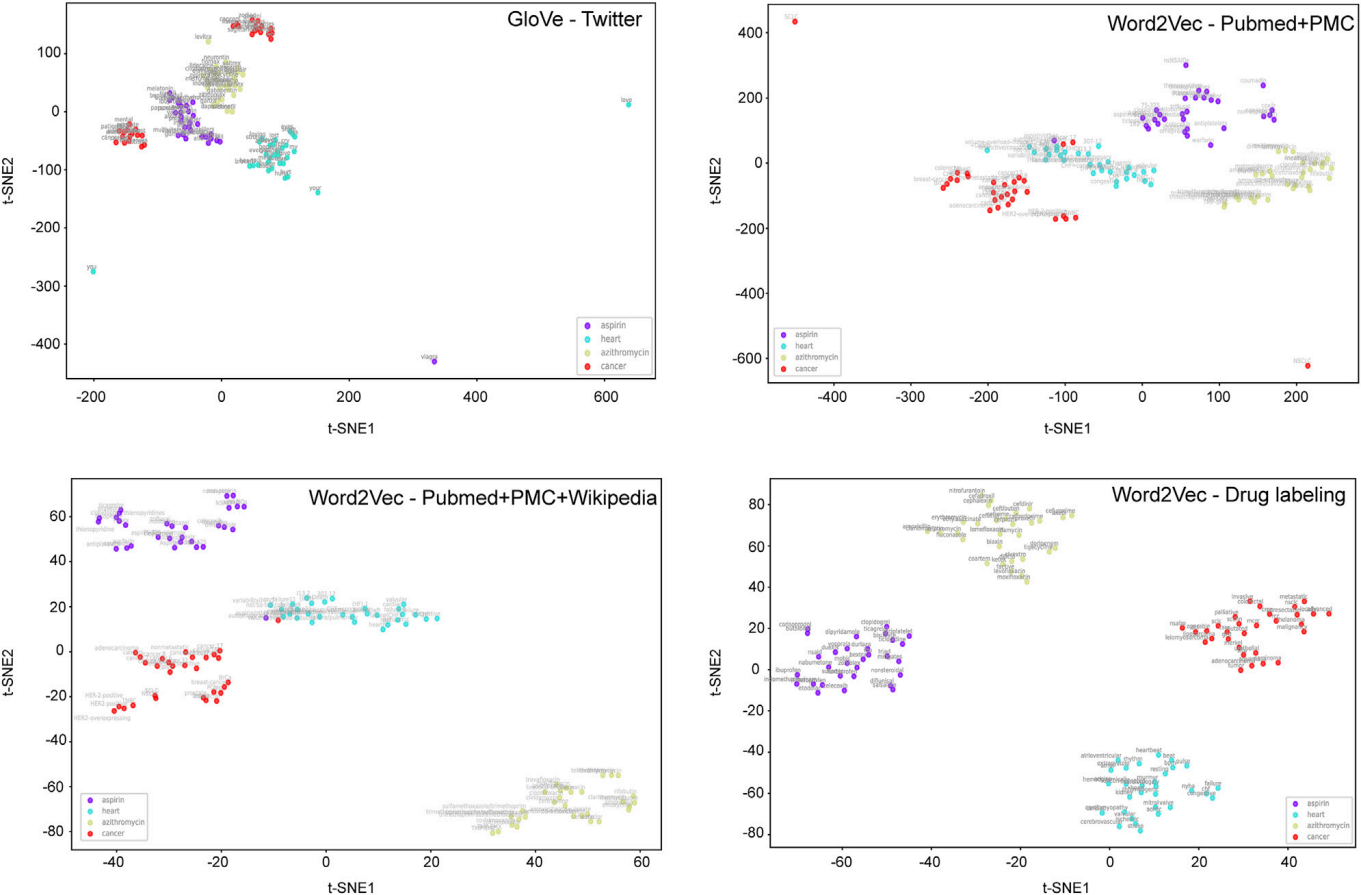
t-SNE analysis of different word embedding models on the queried words.

To develop BiLSTM models for indication classification, we used four types of word embeddings, including Word2vecPubmed + PMC, Word2vecPubmed + PMC + Wikipedia, Word2veclabbeling, and GloVeTwitter. To illustrate the potential benefit of domain-specific embedding, we randomly selected four different domain-specific words (i.e., aspirin, heart, azithromycin, and cancer) to get their top ten most similar words (Figure 2). Figure 3 illustrates the clusters of similar words based on the t-SNE analysis. The Word2veclabbelin, Word2vecPubmed + PMC, and Word2vecPubmed + PMC + Wikipedia models could cluster similar words for the queried words more closely than GloVeTwitter models, highlighting the benefit of domain-specific word embedding for semantic relationship extraction in biomedical applications. We found that the performance of BiLSTM models with domain-specific word embeddings (i.e., MCC = 0.878 for Word2vecPubmed + PMC + Wikipedia > MCC = 0.864 for Word2vecPubmed + PMC > MCC = 0.857 for Word2veclabbeling) was slightly better than that of the BiLSTM model with general domain-based word embedding (MCC = 0.849 for GloVeTwitter). Furthermore, the other 7 performance metrics including accuracies, AUCs, F-scores, sensitivity, specificity, NPV and PPV of domain-specific embedding-based LSTMs were consistently better than general domain embedding-based BiLSTM, indicating domain-specific embedding-based BiLSTMs could extract the indication-related information more accurate (Table 1).

**TABLE 1:** Model performances of nine different AI-based models for indication classification on test set*.

**Models**	**MCC**	**ACC**	**AUC**	**F-score**	**Sensitivity**	**Specificity**	**NPV**	**PPV**
**Bidirectional long short-term memory (BiLSTM)**								
GloVe (twitter)	0.849	0.925	0.981	0.935	0.908	0.950	0.875	0.964
Word2vc (Drug Labeling)	0.857	0.929	0.977	0.940	0.916	0.950	0.883	0.965
Word2vec (PubMed+ PMC)	0.864	0.934	0.977	0.944	0.925	0.946	0.893	0.963
Word2vec (PubMed + PMC+ Wikipedia)	0.878	0.941	0.982	0.950	0.945	0.935	0.921	0.955
**BERT and its derivates**								
DistilBERT	0.820	0.911	0.970	0.922	0.896	0.933	0.862	0.950
ALBERT	0.877	0.941	0.978	0.950	0.964	0.907	0.946	0.937
BERT	0.899	0.951	0.985	0.958	0.949	0.954	0.927	0.968
BioBERT	0.917	0.960	0.987	0.966	0.972	0.943	0.959	0.960
RoBERTa	0.921	0.962	0.987	0.968	0.962	0.962	0.945	0.974

* Positive predictive value (PPV) and negative predictive value (NPV).

### Transformers-Based Models Outperformed the Word Embedding-Based Bidirectional Long Short-Term Memory Model

To further explore the possibility of improving the binary indication classification model performance, we implemented five different fine-turned BERT-like transformer models, including BERT, DistillBERT, ALBERT, RoBERTa, and BioBERT (Table 1). First, all transformer-based models except DistillBERT outperformed word embedding-based BiLSTMs. Second, RoBERTa, BioBERT, and BERT yielded better performance (MCC = 0.921, 0.917, and 0.899, respectively) than the condensed transformers including ALBERT and DistilBERT (MCC = 0.877 for ALBERT and MCC = 0.820 for DistilBERT). Third, domain-specific word embedding-based BiLSTM (i.e., MCC = 0.878 for Word2vecPubmed + PMC + Wikipedia) outperformed the condensed BERT models (i.e., MCC = 0.820 for DistilBERT), highlighting the improvement of model performance based on the large size of the domain-specific corpus, even with the relatively shallow deep learning model. Fourth, the performance of domain-specific BERT (i.e., BioBERT) was comparable to that of RoBERTa, which is trained on top of a large general corpus and with more aggressive hyperparameters.

**FIGURE 4:**
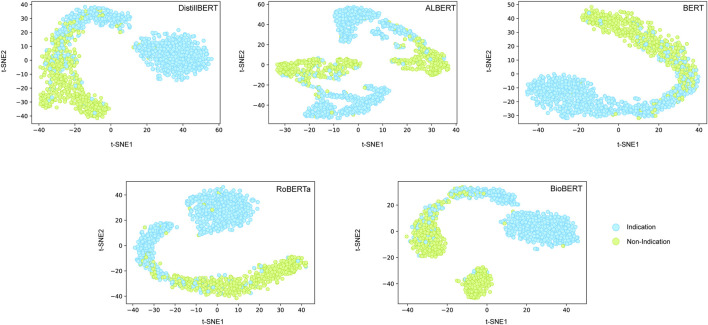
t-SNE analysis of average hidden states of transformers on test set.

We further employed a t-SNE analysis to visualize the contribution of hidden states of transformers on classification performance (Figure 4). We observed the obvious margin for discriminating positives from negatives based on the hidden layer information of most of the transformer models. It is interesting that the positives and negatives samples were closer for the BioBERT model, which may be the reason for the unexpectedly small contribution of domain-specific training for the test set.

### Indication Information Extraction for DrugBank Indication Notes

**FIGURE 5:**
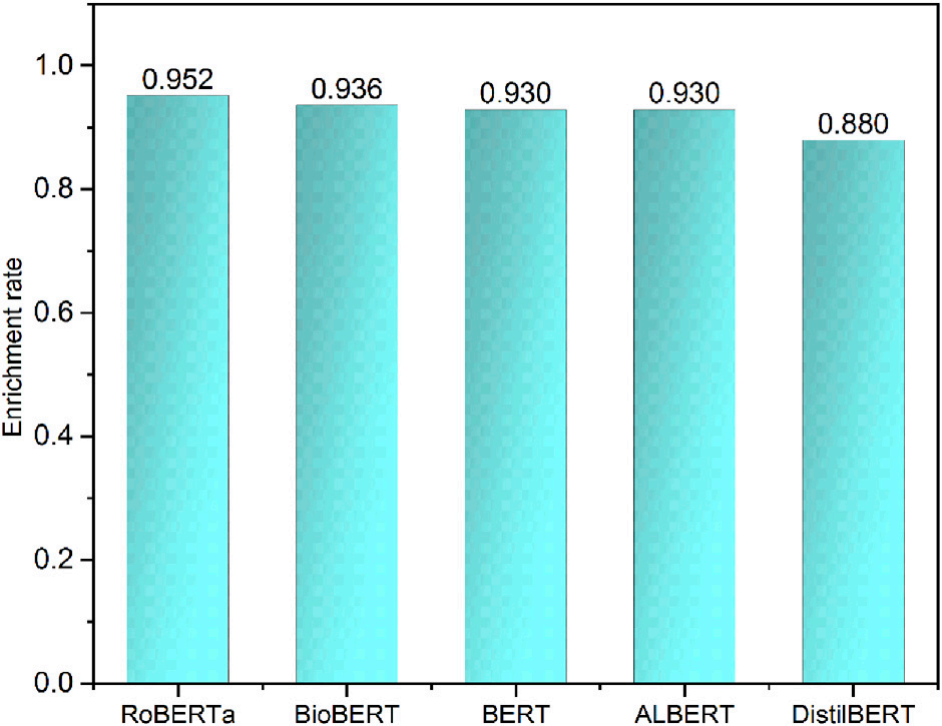
Enrichment rate of fine-tuned transformers on Drugbank indication notes.

Working towards a real-world application, we applied the top performance models to extract the indication-related sentences in DrugBank indication description notes. The Drugbank indication description notes are concise information for drug indications without other information such as contraindications, side effects, and specific population. We considered all drug indication notes as positives. Therefore, we could calculate the enrichment rate that measures the number of indication information sentences correctly recognized by the developed models. The enrichment rates were ranked as RoBERTa (0.952) > BioBERT (0.936) > BERT (0.930), which is consistent with previous results based on test sets (Figure 5). Based on the model performances of both the test set and external validation set, BioBERT and RoBERTa could provide more robust performance and better generalization ability for different data resources.

## Discussion

Drug indications provide key medical information to support clinical decision making and promote the appropriate use of medicines. Furthermore, drug indication information is also considered a fundamental resource to assist in the standardization of medical coding and to potentially eliminate medical errors (Fung et al., 2013). AI-powered NLP models have successfully been applied to various biomedical-related tasks such as biomedical entity recognition, text classification and questioning and answering. However, a standard corpus for domain-specific tasks is urgently needed to advance the development of AI algorithms. In this study, we developed a five-tier based Drug Indication Classification and Encyclopedia (DICE) based on FDA approved drug labels with a consensus manual curation strategy, to facilitate automatic indication information extraction from free text with AI-powered NLP approaches. To verify the utility of the proposed DICE, we conducted a comprehensive comparison of nine deep learning-based NLP models consisting of word embedding-based BiLSTMs and BERT family models. Encouragingly, the top models such as RoBERTa and BioBERT outperformed others with MCCs greater than 0.910 and accuracy greater than 0.960 on test sets, and enrichment rates greater than 0.930 on DrugBank indication notes, demonstrating the great potential of the DICE with AI for automatic indication information identification.

There have been a few attempts to curate the standard corpus of drug indication information for NLP development. However, the sample size is limited (e.g., ∼150 drug labels) (Khare et al., 2015). Here, we used the entire list of FDA approved human prescription drugs to develop the DICE with a five-tier classification scheme. The DICE scheme took into account the FDA guidance requirement for ‘INDICATION AND USAGE’ section drafting (https://www.fda.gov/regulatory-information/search-fda-guidance-documents/indications-and-usage-section-labeling-human-prescription-drug-and-biological-products-content-and). The potential utility of DICE can be divided into two aspects: 1) The DICE could serve as a standard biomedical classification corpus for deep learning-based NLP algorithm development; and 2) the DICE could also be utilized for indication information extraction model development towards real world applications such as off-label use and potential drug repurposing opportunities derived from free-text resources (e.g., PubMed, EMR, patent, and social media).

The benefits of the domain-specific training on different biomedical applications have been discussed elsewhere (Beltagy et al., 2019; Huang et al., 2019; Lee et al., 2020). The domain-specific word embedding-based BiLSTM yielded better prediction performance than those built from general domain corpora. Furthermore, the explainability of domain-specific word embedding was superior as demonstrated by t-SNE analysis. We did not observe any significant improvement of domain-specific transformers (i.e., BioBERT) compared to the original BERTbase and RoBERTabase on the test set, indicating the performance of transformers may be task-specific and data specific. Furthermore, further training of domain-specific transformers (e.g., BioBERT, SciBERT, and ClinicalBERT) on FDA approved drug labeling data may be a potential direction to pursue even better performance, however, it is out of scope of the current study.

Advances in AI in NLP and increased computational power have allowed various transformer-based language models to be developed and successfully used in different downstream tasks (Devlin et al., 2018; Brown et al., 2020). As proof-of-concept of the utility of the developed DICE, we selected the transformers based on the BERT architecture. Other transformer-based models such as Generative Pre-trained Transformer (GPT) 2/3 (Brown et al., 2020), an autoregressive language model, have demonstrated high performance in different NLP tasks, especially in text generation and reading comprehension, which may be worth further investigation for potential performance improvements, even in the indication information classification task. However, the balance between performance, computational cost, and data size must be considered. Based on model results of the test set and DrugBank data set, the condensed models such as DistillBERT and ALBERT could also largely maintain the prediction performance with a more economical usage of computational resources.

The current version of DICE and associated AI-based language models were based on the English language. Further evaluation of other languages will be a great addition to expand the utility of the developed DICE corpus. First, the proposed data curation process of the DICE corpus is reproducible and could be migrated to the documents in other languages. Accordingly, the associated AI-based language models could be developed for drug indication information extraction in other languages. Second, tremendous efforts have been made to language translation powered by AI in the biomedical domain (Liu et al., 2021). For example, Liu et al. proposed a novel cross-lingual biomedical entity linking model among ten typologically diverse languages, which could translate the domain-specific terminology between the languages. By combining the developed biomedical entity linking model, the proposed indication extraction models could be utilized in other languages. However, further investigation and evaluation are strongly recommended.

It is worthwhile to consider some additional studies to further investigate the utility of DICE in different medical applications. First, the model performance of different models was only evaluated based on one test set. Considering the lack of annotated data (i.e., ground truth) in the other resources, we only employed DrugBank indication notes as positives to verify the proposed models for a real-world application. Some extra verifications are strongly recommended for expanding the utility of the developed DICE and accompanying models. Second, the developed DICE and classification models could serve as the first step to extract indication information. The other biomedical entity recognition approaches (e.g., UMLS MetaMap (Aronson, 2001) or BioBERT (Lee et al., 2020)) could be applied to extract disease-related terms for further applications. Third, in the current study, the AI-based indication extraction models are binary-based. Considering the unbalanced distribution of the five defined categories (4,297 indication, 1,673 clinical observations, 701 contraindication, 492 usage instructions, and 68 side effects), we suggest further investigations on the performance of the multi-class models. Lastly, while the developed DICE is a five-tier indication classification scheme, we only investigated its utility for automatic indication information extraction through its usage as a binary classifier. Evaluation for potential utility for testing multiple-class model performance is suggested.

Automatic drug indication extraction is of great importance for different biomedical applications. To fill this gap, we developed the DICE to facilitate AI-based algorithm development and verification. We hope our developed DICE will be considered as a standard drug indication classification corpus, providing the opportunity for other biomedical NLP researchers to promote AI-powered indication extraction in different real-world applications.

## Data Availability

The original contributions presented in the study are included in the article/Supplementary Material, further inquiries can be directed to the corresponding authors.
